# Edge-oriented and steerable hyperbolic polaritons in anisotropic van der Waals nanocavities

**DOI:** 10.1038/s41467-020-19913-4

**Published:** 2020-11-30

**Authors:** Zhigao Dai, Guangwei Hu, Guangyuan Si, Qingdong Ou, Qing Zhang, Sivacarendran Balendhran, Fahmida Rahman, Bao Yue Zhang, Jian Zhen Ou, Guogang Li, Andrea Alù, Cheng-Wei Qiu, Qiaoliang Bao

**Affiliations:** 1 Engineering Research Center of Nano-Geomaterials of Ministry of Education, Faculty of Materials Science and Chemistry, China University of Geosciences, 388 Lumo Road, 430074 Wuhan, P. R. China; 2 Department of Materials Science and Engineering, ARC Centre of Excellence in Future Low-Energy Electronics Technologies (FLEET), Monash University, Wellington Road, Clayton, Victoria 3800 Australia; 3 Department of Electrical and Computer Engineering, National University of Singapore, 4 Engineering Drive 3, Singapore, 117583 Singapore; 4 Photonics Initiative, Advanced Science Research Center, City University of New York, New York, NY 10031 USA; 5 College of Information Science and Engineering, Northeastern University, 110004 Shenyang, China; 6 School of Physics, The University of Melbourne, Parkville, VIC 3010 Australia; 7 School of Engineering, RMIT University, Melbourne, VIC 3000 Australia; 8 Physics Program, Graduate Center, City University of New York, New York, NY 10016 USA; 9 Department of Applied Physics, The Hong Kong Polytechnic University, Hung Hom, Kowloon, Hong Kong, P. R. China

**Keywords:** Metamaterials, Nanocavities, Polaritons

## Abstract

Highly confined and low-loss polaritons are known to propagate isotropically over graphene and hexagonal boron nitride in the plane, leaving limited degrees of freedom in manipulating light at the nanoscale. The emerging family of biaxial van der Waals materials, such as α-MoO_3_ and V_2_O_5_, support exotic polariton propagation, as their auxiliary optical axis is in the plane. Here, exploiting this strong in-plane anisotropy, we report edge-tailored hyperbolic polaritons in patterned α-MoO_3_ nanocavities via real-space nanoimaging. We find that the angle between the edge orientation and the crystallographic direction significantly affects the optical response, and can serve as a key tuning parameter in tailoring the polaritonic patterns. By shaping α-MoO_3_ nanocavities with different geometries, we observe edge-oriented and steerable hyperbolic polaritons as well as forbidden zones where the polaritons detour. The lifetime and figure of merit of the hyperbolic polaritons can be regulated by the edge aspect ratio of nanocavity.

## Abstract (web summary)

The possibility to manipulate the propagation of polaritons is limited by the isotropy of 2D materials like graphene and hexagonal boron nitride. Here, the authors exploit the anisotropy of α-MoO_3_ and study edge tailored hyperbolic polariton manipulation in α-MoO_3_ nanocavities via real space nanoimaging.

## Introduction

One of the main goals of nanophotonics is to manipulate and control light at the nanoscale^1–7^. In van der Waals (vdW) nanomaterials and their layers, the interaction of light with different carriers leads to half-light-half-matter quasiparticles, such as plasmon polaritons in graphene^8–12^, exciton polaritons in semiconductor monolayers^13,14^, and phonon polaritons (PhPs) in polar materials^6,15–22^, which all enable diffraction-less confinement and guiding of light at the nanoscale. In particular, PhPs in polar vdW materials, such as hexagonal boron nitride (hBN) endowed with natural hyperbolic response, offer a low-loss, highly confined and ray-like light propagation, enabling high-quality resonances, hyper-lensing, and nano-imaging^23–26^. This extreme form of optical anisotropy is inherently out of plane, while only recently we have discovered in-plane hyperbolic PhPs in vdW α-MoO_3_^27–29^ and α-V_2_O_5_^30^ layers, which provides an unusual material platform for nanoscale light manipulation.

To further control light with polaritons, we can resort to nanocavities or nanoresonators made of polar vdW materials, offering further enhanced wave confinement and light-matter interactions. For instance, high-quality PhPs exist both in individual hBN nanocones^24^ and arrays of polaritonic antennas;^31^ Fabry–Perot resonances are sustained in hBN ribbons;^15,16,31^ and deep subwavelength confinement is found in polaritonic crystals of hole arrays in hBN thin films thanks to Bloch modes^24^. However, these studies rely on out-of-plane hyperbolic and uniaxial hBN, and cannot be readily applicable for in-plane hyperbolic nanocavities, which are arguably more accessible and practical. Unlike graphene and hBN, in which edges of those planar structures with arbitrary orientation will behave in the same way^2,6^, polaritons in α-MoO_3_ may manifest optical properties that strongly depend on the crystallographic orientation of the edges they interact with, due to the in-plane anisotropic nature. One simple argument is that the orientation of an in-plane edge is not optically distinguishable in uniaxial layered materials with its optical axis perpendicular to the interface. But it may significantly affect optical responses when the line orientation and optical axis are non-orthogonal or unparallel, which can be seen in natural negative refraction in titled anisotropic calcite^32,33^ and may also lead to intuitively exotic but largely unknown polaritonic responses in the case of biaxial and in-plane anisotropic polar vdW materials like α-MoO_3_^27,28^ and V_2_O_5_^30^.

**Fig. 1:**
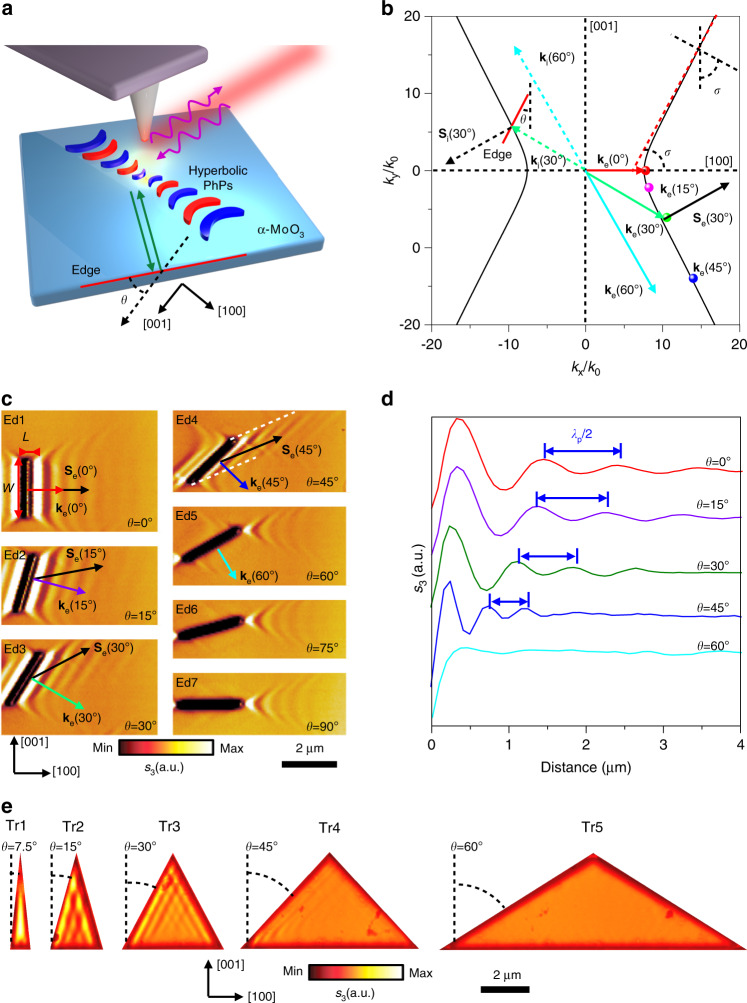
Edge-tailored hyperbolic PhPs in α-MoO_3_. **a** Schematic diagram of edge-tailored PhPs in α-MoO_3_. The edge orientation is defined as angle θ with respect to the [001] direction. Green arrows indicate the incident PhPs waves launched by the laser-illuminated (purple curve arrows) AFM tip and reflected by the edge (red line). **b** Angle-dependent **k**_e_ isofrequency contour of PhPs in α-MoO_3_ at *ω* = 889.8 cm^−1^. The solid lines and points stand for experimental results concluded from Fig. 1c. The green and black dotted arrows illustrate the incidence wavevector **k**_i_ and Poynting vector **S**_i_, respectively. Generally, **k**_i_ and **S**_i_ are non-collinear. The reflected Poynting vector **S**_e_ (solid arrows) is not parallel to the reflected wavevector **k**_e_ (different color solid arrows) but antiparallel to **S**_i_. *σ* is the open angle. **c** Real-space imaging of edge-tailoring PhPs at angle-dependent α-MoO_3_ edges (length *L*: 2.5 µm; width *W*: 200 nm; sample thickness *d*: 210 nm, *L* and *W* defined in the Ed1). **d** s-SNOM line traces along the direction perpendicular to the edges in Ed1-Ed5. **e** Near-field amplitude *s*(*ω*) of PhPs on isosceles triangle α-MoO_3_ nanocavities with bottom edge perpendicular to the [001] crystal direction (height length: 4.33 μm; thickness: *d* = 175 nm); The angles between adjacent sides of the series of triangles with respect to the [001] direction are approximately 7.5°, 15°, 30°, 45°, and 60°, respectively.

Herein, we propose and demonstrate a nontrivial way to manipulate and direct in-plane hyperbolic PhPs via tailoring the edge orientations of a resonant nanocavity with respect to crystallographic directions in biaxial vdW α-MoO_3_ (Fig. 1a). We find that, unlike out-plane hyperbolic PhPs, PhPs parallel to the edge of α-MoO_3_ can only be observed when the edge normal direction is within the open angle of the in-plane hyperbolic isofrequency dispersion. Such exotic phenomenon allows on-demand design of tunable, highly confined, and directional PhP propagation in α-MoO_3_ nanocavities by simply examining their edges. Our work therefore offers rational control of the optical field at the sub-diffraction scale and opens an avenue for engineering PhPs in biaxial polar vdW materials.

## Results

### Edge-tailored hyperbolic PhPs in anisotropic vdW layers

We first investigated the effect of edge orientation on propagating hyperbolic PhPs in real space, resorting to scattering-type scanning field near-field microscopy (s-SNOM, see “Methods”)^27,28^. In our geometry, the edge orientation is represented with the angle θ, defined between the edge and the [001] direction of α-MoO_3_ crystal (Fig. 1a). This angle also indicates the direction of the wavevector, as shown in the isofrequency dispersion (Fig. 1b)^27,28,34^, as the wavevector direction is normal to the edge. We also define the open angle of the hyperbolic dispersion as *σ*. Via a simple geometric argument, it can be seen that when *θ* > *σ*, there is no intersection between the wavevector and the polariton dispersion, hence PhP propagation is forbidden along this direction. In other words, the appearance of PhP fringes parallel to the edge is only allowed when *θ* < *σ*. A more rigorous discussion can be found in Methods. As *θ* changes from 0 to *σ*, the intersection (dots in Fig. 1b) between the wavevector direction (edge normal direction) and the dispersion line moves apart from the original point, suggesting a larger value for the allowed wavevector and a smaller polariton wavelength.

To verify our theoretical prediction, a series of grooves were fabricated using focused ion beam (FIB) in a large-area α-MoO_3_ sample (Supplementary Fig. 1a), with the edge orientation angle (*θ*) varying from 0° to 90° at a step width of 15° (denoted as edge Ed1 to Ed7 in Fig. 1c). The near-field amplitude images near those edges were measured via the scattering-type scanning field near-field microscope (s-SNOM, see methods) at *ω* = 889.8 cm^−1^. A groove can be geometrically decomposed into four components, two end points and two parallel edges. When *θ* = 0° (Ed1, Fig. 1c), the PhPs pattern consists of the interference fringe parallel to the line of the edge and hyperbolic wavefronts at two end points. As *θ* increases from 0° to 45° (Ed1–Ed4, Fig. 1c), the interference fringes parallel to the edge line still exist, while the angle between the propagation direction (Poynting vector **S**_e_, black solid arrow) and the wavevector (**k**_e_, different color solid arrows, perpendicular to the groove) increases. For θ = 45° (Ed4), the two white dashed lines indicates the region that the PhPs are allowed. It is noteworthy that there are no PhPs fringes parallel to the edges (Ed5–Ed7, Fig. 1c) when *θ* ≥ 60°, although the hyperbolic wavefronts preserve at two end points, in agreement with our theoretical prediction. In addition, the spacing of two neighboring fringes reflected to the same side (corresponding to *λ*_p_/2, *λ*_p_ is the PhP wavelength) reduces from 450 nm to 200 nm with a confinement factor from 12.5 to 25.5, as plotted by the line traces in Fig. 1d. The corresponding wavevectors (*k*_e_ = 2π/*λ*_p_) retrieved from the experiments can fit excellently with the intersection points of the wavevector normal to the edge and the dispersion line (Fig. 1b). All these features attest to the impact of the edge and the robustness of our approach to tune the PhPs distribution by simply tailoring the edge orientation of α-MoO_3_.

On this basis, we extend the concept of edge-tailored hyperbolic PhP modes in Fig. 1 to a closed nanocavity composed by the lowest number of edges, i.e. triangular α-MoO_3_ nanocavities. For simplicity, we used isosceles triangle shapes (SEM image shown in Supplementary Fig. 1b). Such triangular shapes are intentionally designed with their bottom edge perpendicular to the [001] crystal direction of α-MoO_3_ (Tr1–Tr5, see the measured near-field signals in Fig. 1e). In general, the polariton distribution of such triangle nanocavities can be regarded as the interference of PhPs reflected from two edges. Firstly, no polariton fringes parallel to the bottom edge are observed, as expected, since this edge is equivalent to Ed7 in Fig. 1c and thus does not contribute to the polariton distribution in the nanocavity. In sharp contrast, this property does not exist in the similar triangular nanocavities made of graphene^35^ or hBN (see our simulations in Supplementary Fig. 3a and Supplementary Notes 2 and 4), because of their in-plane isotropy: in such geometries we always find polaritons reflected from every edge. Secondly, when the apex angle of the triangle increases, the edge orientation angle *θ* of their equal sides accordingly grows. The polariton distribution determined by the oblique sides is expected to be very small when the edge orientation angle *θ* (Tr5) is larger than the open angle, and the whole triangle manifests a forbidden zone without polaritonic signature, a feature that can be traced back to Fig. 1c (Ed5). More verifications of such triangular shapes with a bottom edge along the [100] crystal directions can be found in Supplementary Fig. 3b, which further validates our discussions. Our experimental results are also corroborated with full-wave simulations using finite-difference time domain method (see Supplementary Fig. 4 and Supplementary Note 3). Hence, we can conclude that the polariton distribution within a geometric nanocavity strongly depends on the edge orientation in α-MoO_3_.

### Edge-tailored hyperbolic PhPs in anisotropic vdW square nanocavities

**Fig. 2:**
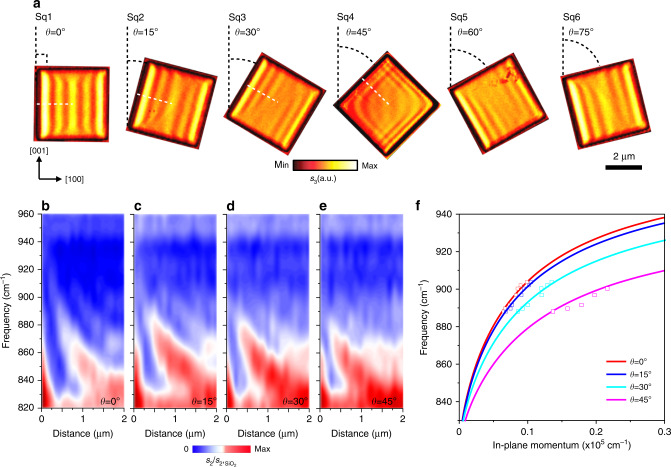
Edge-tailored PhPs in square α-MoO_3_ nanocavities. **a** Real-space imaging of edge-tailored PhPs in rotated square nanocavities (side length: 4 μm; thickness: *d* = 175 nm) which was fabricated in the same piece of α-MoO_3_ sample. **b**–**e** Nano-FTIR spectral line scans perpendicular to the rotated edges (white dotted lines in Sq1–Sq4), showing the near-field amplitude *s*_2_(*ω*) (normalized to the near-field amplitude on the SiO_2_ substrate, *s*_2,SiO2_(*ω*)) as a function of distance between tip and edge. Color dashed lines are guides for the eye of signal maxima. **f** Dispersion relation of hyperbolic PhPs in square α-MoO_3_ nanocavities. The red, blue, cyan and pink line corresponds to the theoretical dispersions at different rotation angles *θ* = 0°, 15°, 30°, and 45° respectively. The squares correspond to the experimentally measured data.

We next proceed to study a more complex geometry, i.e., a rectangle. A series of square shape nanocavities were fabricated with varying rotation angles with respect to the [001] direction (SEM image in Supplementary Fig. 1c). We see that anisotropic polaritons are regulated by the boundary edges and form angle-dependent patterns (Fig. 2a). Interestingly, the PhP fringes of a pair of square nanocavities with rotation angles *θ*_1_ and *θ*_2_ appear to be strictly mirror-symmetrical, when *θ*_1_ + *θ*_2_ = 90°, for example, *θ*_1_ = 15 or 30° (square Sq2 or Sq3, Fig. 2a) and *θ*_2_ = 75 or 60° (square Sq6 or Sq5, Fig. 2a). Specifically, for *θ* = 45° (square Sq2, Fig. 2a), hyperbolic PhPs are equally generated at left and right edges and interference at the top and bottom vertices, similar to those in the corner of graphene and hBN described in Supplementary Note 4 and Supplementary Fig. 6, as every edge contributes to the polariton distribution in the nanocavity. Due to the interference of the hyperbolic wavefronts from the two horizontal corners of the square, an eye shape pattern at the center of the square α-MoO_3_ nanocavity can be observed. These features strongly underpin the opportunity offered by in-plane anisotropic nanocavities based on α-MoO_3_. To further corroborate the observation of angle-dependent PhPs, we performed nanoscale Fourier-transform infrared spectroscopy (nano-FTIR, “Methods”) measurements perpendicular to the edges with varied angles (as the **k**_e_ direction, white dotted lines in Fig. 2a), as shown in Fig. 2b–e. As evidence of the edge effect, a series of angle-dependent signal maxima (color dashed lines, Fig. 2b–e), corresponding to phonon frequencies of α-MoO_3_, are observed within the band limits. We find that the signal maxima show different spacings (corresponding to *λ*_p_) and that *λ*_p_ decreases not only with a frequency increase, but also with increasing angle *θ*. The experimental and numerical dispersion relations of PhPs in α-MoO_3_ with *θ* = 0°, 15°, 30°, and 45° are compared, showing good consistency (Fig. 2f, see more details “Methods”). Overall, these nano-FTIR results and dispersion relations confirm that the rotating square α-MoO_3_ nanocavities support edge-tailored PhPs with in-plane anisotropic propagation, thus adding another strategy to the library of nano-light manipulation in vdW materials.

### Edge-tailored hyperbolic PhPs in anisotropic vdW rectangle nanocavities with different aspect ratios and rotation angles

**Fig. 3:**
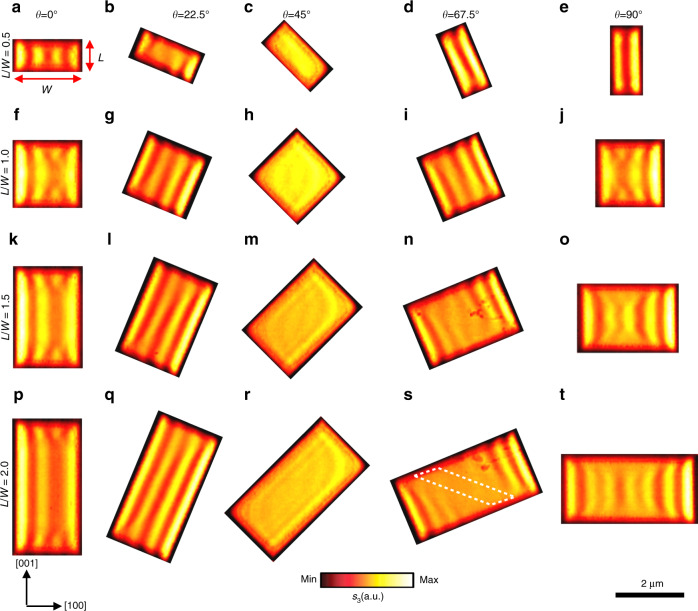
Edge-tailored PhPs in rectangle α-MoO_3_ nanocavities with different aspect ratios and rotation angles. Nano-infrared imaging of near-field amplitude *s*(*ω*) in rectangle α-MoO_3_ nanocavities with different aspect ratios (*L*/*W*, on the left) and angle *θ* (on the top). The length *L* and width *W* are defined in the panel **a**. The width *W* of each rectangle is 2 μm and the thickness is 155 nm. The length *L* of each rectangle are 1 μm (**a**–**e**), 2 μm (**f**–**j**), 3 μm (**k**–**o**), and 4 μm (**p**–**t**), respectively. All the images were measured at frequency *ω* = 896.9 cm^−1^. The white dotted parallelogram in panel **s** indicates the forbidden zone of PhPs waves.

We then change the edge aspect ratio *L*/*W* (*L* and *W* are the length and width, respectively) of the rectangle, at different rotation angles, as presented in Fig. 3. Dramatically different from linear hBN antennas^16^ and rectangular hBN nanocavities (see Supplementary Fig. 7 and Supplementary Note 4) with in-plane isotropy, directional polaritonic propagation can be clearly observed in α-MoO_3_ nanocavities, other than bidirectional propagation with cross-coupled interference. The square nanocavities (*L*/*W* = 1) in the second row (Fig. 3f–j) have smaller size compared with those in Fig. 2a (square Sq2 to Sq6), but they show similar mirror-symmetric features as discussed above. When the edge aspect ratio *L*/*W* is reduced to 0.5 (Fig. 3a–e), the polariton fringes show obvious characteristics of a Fabry–Perot cavity at small rotation angles (*θ* = 0° and 22.5°). As the angle increases to *θ* = 67.5° and 90°, the near-field pattern changes into two bright fringes due to the reflection of long edges. The eye shape pattern is compressed along the long edge while *θ* = 45°. When the edge aspect ratio becomes larger than 1 (*L*/*W* = 1.5 in Fig. 3k–o and *L*/*W* = 2 in Fig. 3p–t), the near-field pattern changes for different rotation angles. At a rotation angle *θ* = 22.5°, the polariton fringes are prolonged along the long edge while the number of fringes does not change (Fig. 3l, q). At the rotation angle *θ* = 45°, the eye shape pattern at the center is elongated along the long edge direction (Fig. 3m, r). By contrast, more fringes can be observed at large rotation angle *θ* = 67.5° (Fig. 3n, s). It is interesting to see a parallelogram shape zone (white dotted frame) without fringes in Fig. 3s. Such area can be considered a forbidden zone unreachable to the Poynting vector **S**_e_ of the PhPs formed on the left and right sides. The asymmetry fringes in the Fig. 3f, k, p are attributed to the superposition effect^36^ and the slightly lopsided, pyramidal atomic force microscope (AFM) tip^37^. In order to corroborate the experimental observations, we performed full-wave numerical electromagnetic simulation to restore the near-field images (Supporting Note 3). The simulation result of a typical example with edge aspect ratio 1.5 is displayed in Supplementary Fig. 5a–e, which shows a good consistency with experimentally measured near-field images (Fig. 3k–o). We ascribe those extraordinary modal near-field patterns in the confined geometry of rectangular nanocavities to the generation of directional guided PhPs in α-MoO_3_ single crystals, unlike conventional isotropic polaritons^2,14,16^.

### Lifetimes and FOM of hyperbolic PhPs in anisotropic vdW nanocavities synergistically regulated by aspect ratio and frequency

**Fig. 4:**
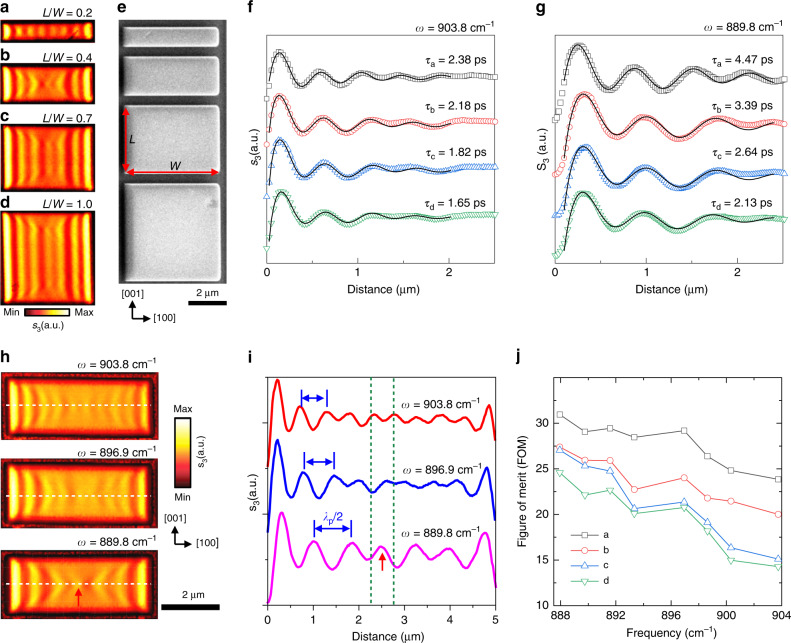
Lifetimes and FOM of hyperbolic PhPs synergistically regulated by aspect ratio and frequency. **a**–**d** Near-field images of large rectangular α-MoO_3_ nanocavities with different aspect ratios. The length *L* = 1 μm, 2 μm, 3.5 μm, and 5 μm, respectively. *L* along the [001] direction and *W* along the [100] direction are defined in panel **e**. The width *W* is 5 μm and the thickness *d* is 175 nm. *ω* = 896.9 cm^−1^. **e** SEM image of nanocavities in panel **a**–**d**. **f**, **g** Lifetime of hyperbolic PhPs in α-MoO_3_ nanocavities with different aspect ratios at *ω* = 903.75 cm^−1^ and 889.8 cm^−1^, respectively. s-SNOM line traces along the [100] direction of the nanocavities (color hollow patterns) in Fig. 4a–d, respectively. Damped sine-wave functions (black solid lines) were fitted to the data. **h** Nano-infrared images of near-field amplitude *s*(*ω*) in rectangle α-MoO_3_ nanocavities at the frequencies 903.8 cm^−1^, 896.9 cm^−1^, and 889.8 cm^−1^, respectively. The length *L*, width *W* and thickness *d* of rectangle are 5 μm, 2 μm and 155 nm, respectively. **i** s-SNOM line traces along rectangle α-MoO_3_ nanocavities shown in (**h**). **i**, s-SNOM line traces along rectangle α-MoO_3_ nanocavities shown in (**h**). Red arrow and green dashed lines are guides for the eye of hyperbolic hot-spot in the image and corresponding s-SNOM line trace. **j** FOM of rectangular α-MoO_3_ nanocavities in panel (**a**–**d**) as a function of frequency.

It is also interesting to find that the edge aspect ratio can regulate the lifetime and figure of merit (FOM) of the nanocavity polariton. The lifetime *τ* = *L*/*v*_g_ and FOM *Q* = Re(*q*)/Im(*q*) can be used to characterize the loss features of polariton propagation^38^ (Supplementary Note 5). A set of large rectangle nanocavities with different edge aspect ratios were fabricated along the [001] direction, as shown in Fig. 4a–e. The obtained amplitude lifetime of PhPs is approximately 1.5 times as in the narrowest nanocavity (*L*/*W* = 0.2, Fig. 4a, *τ*_a_ = 2.38 ± 0.08 ps) than in the square nanocavity (*L*/*W* = 1, Fig. 4d, *τ*_d_ = 1.65 ± 0.03 ps) as shown in Fig. 4f. Furthermore, the narrowest rectangular nanocavity has a better FOM in the 888 to 904 cm^−1^ with an improvement from about 1.3 to almost twice of that in the square α-MoO_3_ nanocavity (see Fig. 4j). For the in-plane hyperbolic PhPs in our α-MoO_3_ nanocavities, the narrower nanocavity is conducive to confine the energy in the [100] direction and match with the propagation direction and the Poynting vector direction, resulting in a longer lifetime and larger FOM.

Finally, the edge-tailored polariton can be effectively modulated via the frequency, since the open angle is dispersive. The near-field images of rectangular nanocavity are displayed in Fig. 4h, revealing that the PhP wavelength decreases from 1484 nm to 1026 nm as the excitation frequency increases from 889.8 to 903.8 cm^−1^ (Fig. 4i). Interestingly, when the edges match the appropriate edge aspect ratio (*L*/*W* = 0.4), we clearly observe a hyperbolic hot-spot (red arrow and green dashed lines) at the center of the nanocavity (889.8 cm^−1^, Fig. 4h, i). The corresponding absolute value of the Fourier transform of the near-field images in Fig. 4h are presented in Supplementary Fig. 8, which conforms to analytical dispersions of in-plane hyperbolic sheets^28,34^. Besides, the open angle of the hyperbola gradually increases when the working frequency increases, as the in-plane anisotropy becomes stronger. For the same reason, as the frequency decreases and so does the open angle, the PhP amplitude lifetime of narrowest nanocavity is increased to approximately 4.5 ps (twice higher than that of PhPs in α-MoO_3_^28^ flake at the same frequency and isotopically enriched hBN flake^38^) as shown in Fig. 4g. In combination with other regulation methods except the frequency, such as the strain^39^, ion intercalation^40^ or formation of array^31^, we envisage the edge-tailored polaritonic behaviors can be further tuned or optimized.

## Discussion

In conclusion, we have demonstrated edge-tailored polaritonic nanocavities by shaping α-MoO_3_. By combining numerical simulations, a rich variety of coexisting polaritonic patterns is found in α-MoO_3_ nanocavities. Through careful design and engineering, directional, steerable and guided hyperbolic PhPs can be achieved and controlled by the shapes, orientations, the in-plane anisotropy, and the vertex angle of tapered α-MoO_3_ nanocavities, which could be potentially applied in miniaturized nanophotonic or energy harvesting devices in the middle infrared regions. Originated from the disorientation between the Poynting vector and the wavevector, the reflection of polaritons from the edge of the biaxial vdW materials is far more complicated than ever in isotropic materials. Due to the limit of the existing imaging technique where launching and probing happen at the same place, it might call on more efforts in the near future, both theoretically and experimentally, to reveal more ubiquitous phenomena of the polaritonic reflection and confinement in a nanocavity made of such in-plane anisotropic materials. Hybridization of edge-tailored PhPs and plasmons may also be incorporated with other exotic optical platforms in the phase change materials or twisted bilayer α-MoO_3_. Our work opens avenues for designer manipulation of hyperbolic PhPs and paves the way for promising applications in metamaterials, nanophotonics and quantum optics based on natural vdW materials.

## Methods

### α-MoO_3_ crystals preparation

Bulk α-MoO_3_ crystals were produced via the sublimation of MoO_3_ powder, followed by the vapor deposition of stratified crystals onto a substrate at a predetermined temperature. In our method, 100 mg of the MoO_3_ powder (Sigma-Aldrich) was weighed and placed at the center of a horizontal furnace at 785 °C. The substrates were placed at a certain distance from the central hot spot at a temperature of approximately 560 °C^41^. And thermal deposition was carried out using a carrier gas (argon, 200 sccm) at 1 Torr. Deposition took place for 1 h and the evaporation temperature was increased slowly at the rate of 5 °C per minute and cooled at the same rate after the procedure. Mechanical exfoliation of MoO_3_ crystals was conducted using polydimethylsiloxane (PDMS) as an alternative to the conventional scotch-tape^42^. Firstly, 300 nm SiO_2_/Si substrates were cleaned using acetone and isopropyl alcohol, followed by drying with compressed N_2_. Subsequently, the substrates were subjected to 10 min of oxygen plasma treatment, ensuring all organic residues are completely removed. The MoO_3_ crystals were then cleaved using flexible pieces of PDMS and transferred onto the SiO_2_/Si substrates. Crystals of interest were identified via optical contrast spectroscopy and their thicknesses were verified using atomic force microscopy.

### Nanocavities fabrication

Focused ion beam (Helios NanoLab™ DualBeam™ microscope, FEI Company) was used to define patterns. All patterns were milled in parallel instead of serially to minimize the re-deposition effect. Subsequently, the patterned samples were annealed at 300 °C for 3 h. The SEM images of the patterned samples are shown in Supplementary Fig. 1.

### s-SNOM and nanoFTIR

The mid-infrared nano-imaging and nano-FTIR spectroscopy were performed at a commercial scattering-type scanning near-field optical microscope (s-SNOM, NeaSpec GmbH, Germany). Metallized, cantilevered atomic force microscope (AFM) tips (Nanoworld, ARROW-NCPt-50) are used as scattering near-field probes in the tapping mode. The AFM tapping frequency and amplitude are approximately 275 kHz and 75 nm, respectively. These laser sources are tunable quantum cascade lasers. When the Pt-covered Si tip is illuminated by the source, the tip will concentrate the incident field into a nanoscale spot at the apex and thus function as a source and probe to resolve the polaritonic effects. More specifically, the local field carrying the information of phase and amplitude are collected at the tip, revealing the interference pattern of forward and backward propagating PhPs. The backscattered radiation is recorded simultaneously with the topography, amplitude and phase near-field images. The backscattered signal by the tip is registered by pseudoheterodyne interferometric detection and then demodulated at the *Ω*-th harmonics of the tapping frequency yielding background free images. In this work, we chose *Ω* = 3. During the s-SNOM experiments, we can unambiguously and in situ assign the [100] and [001] direction of the crystal to the direction of preferential propagation and absent of α-MoO_3_ PhPs in the lower Reststrahlen bands (820 cm^−1^ to 963 cm^−1^), respectively^28^. For nano-FTIR spectroscopy, the Au coated AFM tip was illuminated by a broadband super-continuum laser, and the tip-scattered light was recorded with an asymmetric Fourier transform spectrometer^23,28,30^.

### Dispersion relation of hyperbolic PhPs at arbitrary azimuthal angles in α-MoO_3_

It is very important to calculate the dispersion at an arbitrary azimuthal angle, that is defined as cos*θ* = *k*_x_/*k*_e_. In the dispersion contour shown in Fig. 2f, the analytical dispersion is shown by the solid lines, and the experimentally measured wavevectors (*k*_e_) are represented by the dots, which were taken by measuring the fringe period. The analytical dispersion is found by^43^

1 $$k_{\mathrm{e}} = \frac{{\Psi}}{d}\left[ {{\mathrm{atan}}\left( {\frac{{\varepsilon _1}}{{\varepsilon _{\mathrm{z}}}}{\Psi}} \right) + {\mathrm{atan}}\left( {\frac{{\varepsilon _3}}{{\varepsilon _{\mathrm{z}}}}{\Psi}} \right) + l{\it{\uppi }}} \right],$$

where ${\Psi} = {\rm{i}}\sqrt{\frac{{\varepsilon _z}}{{\varepsilon _x{\cos}^2\theta + \varepsilon _y{\sin}^2\theta }}}$, and *l* denotes the order of polaritonic modes; *ε*_1_ and *ε*_3_ are the permittivity of substrate (SiO_2_) and superstrate (air), respectively. The material property of α-MoO_3_ is adopted from Ref. ^7^.

From this analytical dispersion, we note the in-plane momentum is strongly dependent on the orientation angle, which is different from the in-plane isotropic graphene and hBN. Secondly, in the frequency of interest (835 cm^−1^–950 cm^−1^), *ε*_z_ > 0 and k_e_ is only allowed when Ψ is a real value number which requires *ε*_x_cos^2^*θ* + *ε*_y_sin^2^*θ* < 0, that is $\tan \theta\, < \sqrt { - \frac{{\varepsilon _{\mathrm{x}}}}{{\varepsilon _{\mathrm{y}}}}} = \tan \sigma$, i.e., |*θ*| < *σ*. This derivation justifies our discussion on the intersection of wavevector in the isofrequency contours in Fig. 1b, Supplementary Fig. 2 and Supplementary Note 1. This also agrees with our argument and discussions in the main text that the orientation of the edges could control the appearance and disappearance of the polaritonic fringe parallel to the edge.

## Supplementary information

Supplementary Information

## Data Availability

The data that support the findings of this study are available from the corresponding author upon reasonable request.

## Code availability

The codes that are used to generate results in the paper are available from the corresponding author upon reasonable request.

## References

[1] Dai Z (2020). Artificial metaphotonics born naturally in two dimensions. Chem. Rev..

[2] Nikitin A (2016). Real-space mapping of tailored sheet and edge plasmons in graphene nanoresonators. Nat. Photon.

[3] Fei Z (2015). Edge and surface plasmons in graphene nanoribbons. Nano Lett.

[4] Xu Q (2017). Effects of edge on graphene plasmons as revealed by infrared nanoimaging. Light Sci Appl.

[5] Tamagnone M (2018). Ultra-confined mid-infrared resonant phonon polaritons in van der Waals nanostructures. Sci. Adv..

[6] Chaudhary K (2019). Engineering phonon polaritons in van der Waals heterostructures to enhance in-plane optical anisotropy. Sci. Adv..

[7] Alvarez-Perez G (2020). Infrared permittivity of the biaxial van der Waals semiconductor alpha-MoO_3_ from near- and far-field correlative studies. Adv. Mater..

[8] Ni GX (2018). Fundamental limits to graphene plasmonics. Nature.

[9] Fei Z (2012). Gate-tuning of graphene plasmons revealed by infrared nano-imaging. Nature.

[10] Chen J (2012). Optical nano-imaging of gate-tunable graphene plasmons. Nature.

[11] Jiang T (2019). Ultrafast coherent nonlinear nanooptics and nanoimaging of graphene. Nat. Nanotechnol..

[12] Hu H (2019). Gas identification with graphene plasmons. Nat. Commun..

[13] Hu G (2019). Coherent steering of nonlinear chiral valley photons with a synthetic Au–WS_2_ metasurface. Nat. Photon.

[14] Hu F (2017). Imaging exciton–polariton transport in MoSe_2_ waveguides. Nat. Photon..

[15] Dolado I (2020). Nanoscale guiding of infrared light with hyperbolic volume and surface polaritons in van der Waals material ribbons. Adv. Mater..

[16] Alfaro-Mozaz FJ (2017). Nanoimaging of resonating hyperbolic polaritons in linear boron nitride antennas. Nat. Commun..

[17] Li P (2017). Optical nanoimaging of hyperbolic surface polaritons at the edges of van der Waals Materials. Nano Lett.

[18] Hu, G. et al. Phonon polaritons and hyperbolic response in van der Waals Materials. *Adv. Opt. Mater.* **8**, 1901393 (2019).

[19] Alfaro-Mozaz FJ (2019). Deeply subwavelength phonon-polaritonic crystal made of a van der Waals material. Nat. Commun..

[20] Li P (2018). Infrared hyperbolic metasurface based on nanostructured van der Waals materials. Science.

[21] Hu D (2017). Probing optical anisotropy of nanometer-thin van der waals microcrystals by near-field imaging. Nat. Commun..

[22] Li P (2020). Collective near-field coupling in infrared-phononic metasurfaces for nano-light canalization. Nat. Commun..

[23] Dai S (2014). Tunable phonon polaritons in atomically thin van der Waals crystals of boron nitride. Science.

[24] Caldwell JD (2014). Sub-diffractional volume-confined polaritons in the natural hyperbolic material hexagonal boron nitride. Nat. Commun..

[25] Woessner A (2015). Highly confined low-loss plasmons in graphene-boron nitride heterostructures. Nat. Mater..

[26] Li P (2015). Hyperbolic phonon-polaritons in boron nitride for near-field optical imaging and focusing. Nat. Commun..

[27] Hu G (2020). Observation of topological polaritons and photonic magic angles in twisted van der Waals bi-layers. Nature.

[28] Ma W (2018). In-plane anisotropic and ultra-low-loss polaritons in a natural van der Waals crystal. Nature.

[29] Zheng Z (2018). Highly confined and tunable hyperbolic phonon polaritons in Van Der Waals semiconducting transition metal oxides. Adv. Mater..

[30] Taboada-Gutiérrez J (2020). Broad spectral tuning of ultra-low-loss polaritons in a van der Waals crystal by intercalation. Nat. Mater..

[31] Autore M (2018). Boron nitride nanoresonators for phonon-enhanced molecular vibrational spectroscopy at the strong coupling limit. Light: Sci. Appl..

[32] Chen X (2005). Negative refraction: an intrinsic property of uniaxial crystals. Phys. Rev. B.

[33] Liu Z, Lin ZF, Chui ST (2004). Negative refraction and omnidirectional total transmission at a planar interface associated with a uniaxial medium. Phys. Rev. B.

[34] Hu G (2020). Moiré hyperbolic metasurfaces. Nano Lett.

[35] Zheng ZB (2017). Tailoring of electromagnetic field localizations by two-dimensional graphene nanostructures. Light: Sci. Appl.

[36] Hu F (2017). Imaging the localized plasmon resonance modes in graphene nanoribbons. Nano Lett.

[37] Giles AJ (2016). Imaging of anomalous internal reflections of hyperbolic phonon-polaritons in hexagonal boron nitride. Nano Lett.

[38] Giles AJ (2018). Ultralow-loss polaritons in isotopically pure boron nitride. Nat. Mater..

[39] Huber AJ (2009). Infrared nanoscopy of strained semiconductors. Nat. Nanotechnol..

[40] Wu Y (2020). Chemical switching of low-loss phonon polaritons in α-MoO_3_ by hydrogen intercalation. Nat. Commun..

[41] Balendhran S (2013). Enhanced charge carrier mobility in two-dimensional high dielectric molybdenum oxide. Adv. Mater..

[42] Rahman F (2017). Two-dimensional MoO_3_ via a top-down chemical thinning route. 2D Materials.

[43] Álvarez-Pérez G (2019). Analytical approximations for the dispersion of electromagnetic modes in slabs of biaxial crystals. Phys. Rev. B.

