# Supplementary material for: Edge-oriented and steerable hyperbolic polaritons in anisotropic van der Waals nanocavities
Source: Nat Commun. 2020 Nov 30;11:6086. doi: 10.1038/s41467-020-19913-4 (PMC7705012; doi:10.1038/s41467-020-19913-4)
Supplement: Supplementary file 1 — Supplementary Information [file 41467_2020_19913_MOESM1_ESM.pdf]

Supplementary information for

Edge-oriented and steerable hyperbolic polaritons in anisotropic van der Waals nanocavities

Dai and Hu et al.

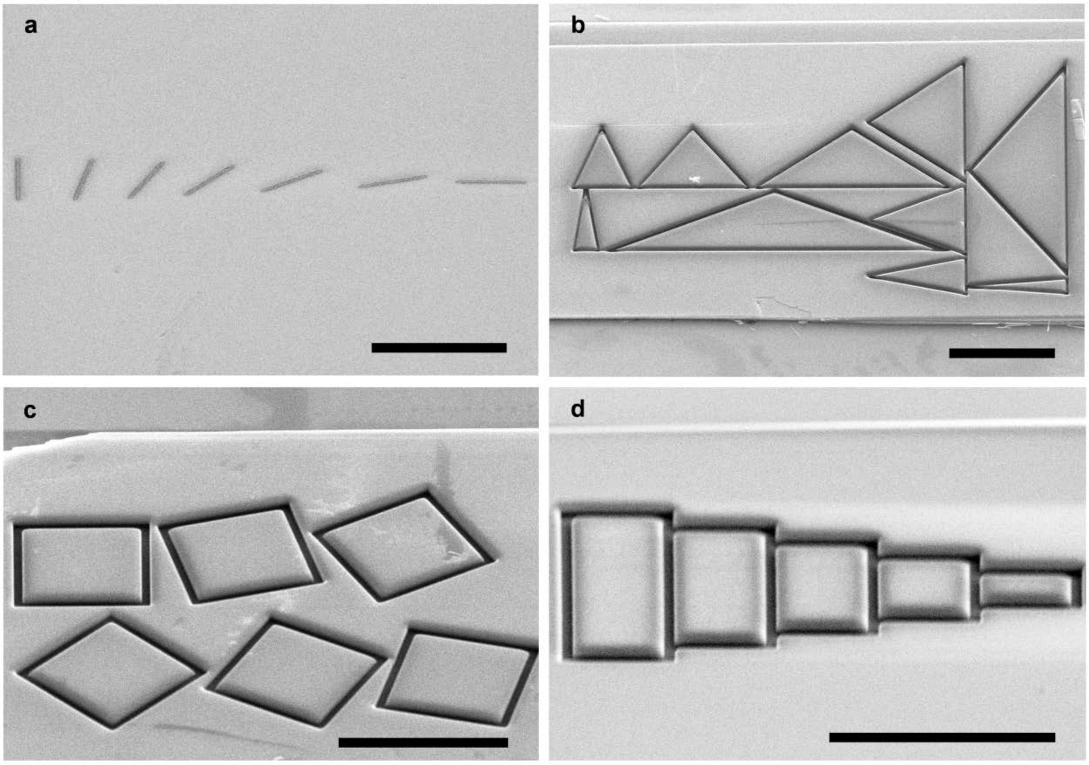

Supplementary Figure 1. **SEM images of α-MoO_3 designs**. Side view of SEM images of **a**, α-MoO_3 edges; **b**, triangular α-MoO_3 nanocavities; **c**, square α-MoO_3 nanocavities; **d**, one sets of rectangle α-MoO_3 nanocavities after FIB fabrication at 52° stage tilt. Scale bar, 5 μm.

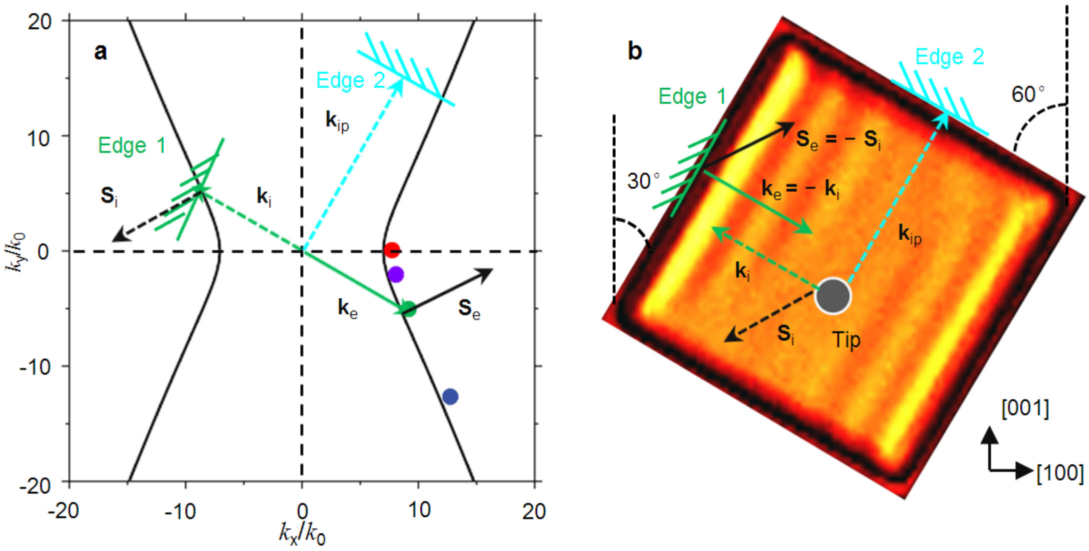

Supplementary Figure 2. **Reflection of hyperbolic PhPs at the edge of α-MoO_3 nanocavities. a,** α-MoO_3 PhPs isofrequency contour at ω = 889.8 cm^{-1}. The green and black dotted arrows illustrate the non-collinearity of wavevector $\mathbf{k}_i$ and Poynting vector $\mathbf{S}_i$. Due to the hyperbolic isofrequency contour, the reflected Poynting vector $\mathbf{S}_e$ (black solid arrow) is not parallel to reflected wavevector $\mathbf{k}_e$ but antiparallel to $\mathbf{S}_i$. **b**. Schematics of a wave back-reflection in square α-MoO_3 nanocavities.

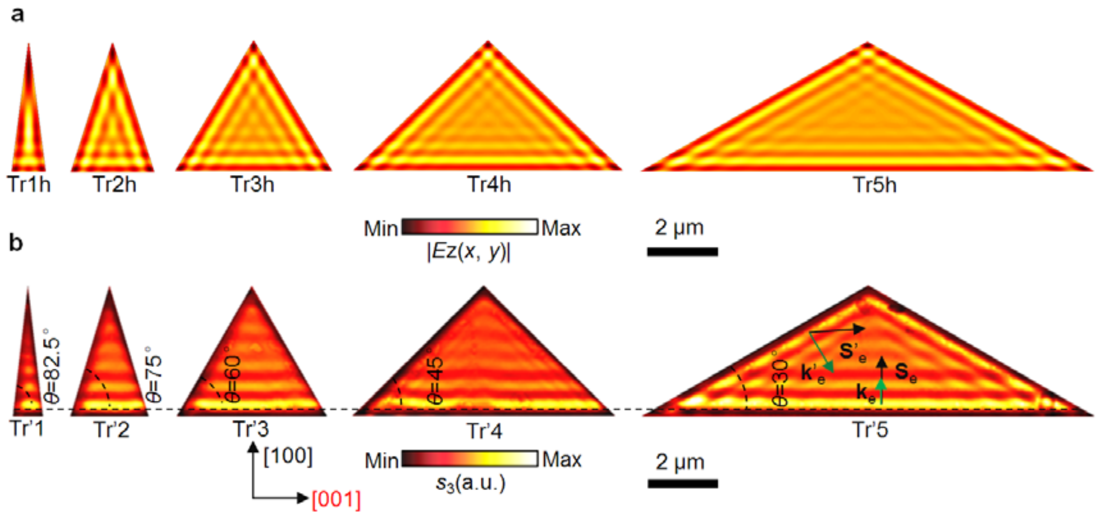

Supplementary Figure 3. **Isotropic PhPs in triangular hBN nanocavities and edge-tailored PhPs in triangular α-MoO_3 nanocavities. a**, The isotropic polariton distribution of triangular hBN nanocavities. The geometric dimensions in **a** are the same as the Fig. **1e** in main text and Supplementary Figure 3**b**. **b**, Near-field amplitude $s(\omega)$ of PhPs on isosceles triangles α-MoO_3 nanocavities with bottom edge parallel to the [001] crystal direction (height length: 4.33 μm; thickness: d = 175 nm); The angles between adjacent sides of the series of triangles with respect to the [001] direction are approximately 82.5°, 75°, 60°, 45°, and 30°, respectively. Poynting vector $\mathbf{S}_e$ and wavevector $\mathbf{k}_e$ reflected from base edges, Poynting vector $\mathbf{S'}_e$ and wavevector $\mathbf{k'}_e$ reflected from adjacent edges of vertex angles.

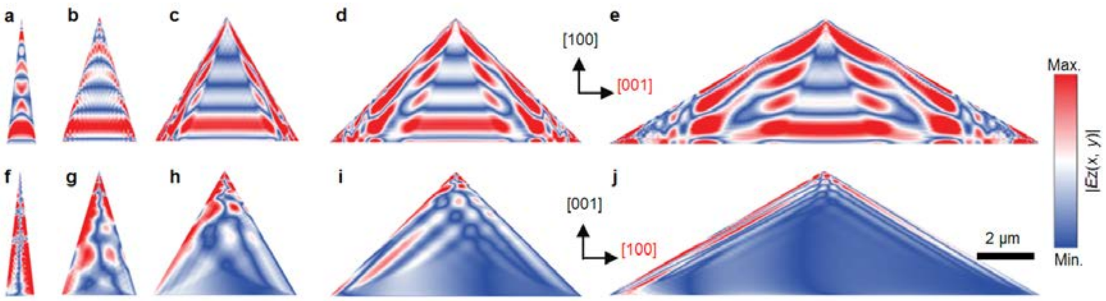

Supplementary Figure 4. **The anisotropic polariton distribution of triangle α-MoO_3 nanocavities**. **a-e** and **f-j** calculated near-field amplitude images $|E_z(x, y)|$ corresponding to triangular α-MoO_3 in Fig. Supplementary Figure 3b and Fig. 1**e** at ω = 889.7 cm^{-1} (colour key at right). Panel **a-e** are obtained under the normal incidence, and **f-j** are under oblique incidence with incidence angle 30 degrees.

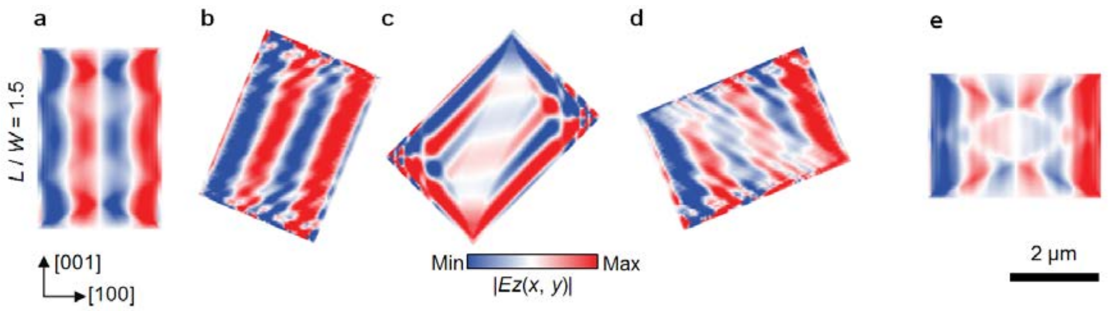

Supplementary Figure 5. The anisotropic polariton distribution of rectangle α-MoO_3 nanocavities. **a-e,** calculated near-field amplitude images $|E_z(x,y)|$ corresponding to α-MoO_3 rectangles in Figs. 3 **k-o** (colour key below) at ω = 896.9 cm^{-1}. Near-field distributions are obtained via the electromagnetic wave excitation in α-MoO_3 nanocavities with θ = 0, 22.5, 45, 67.5, and 90°, respectively.

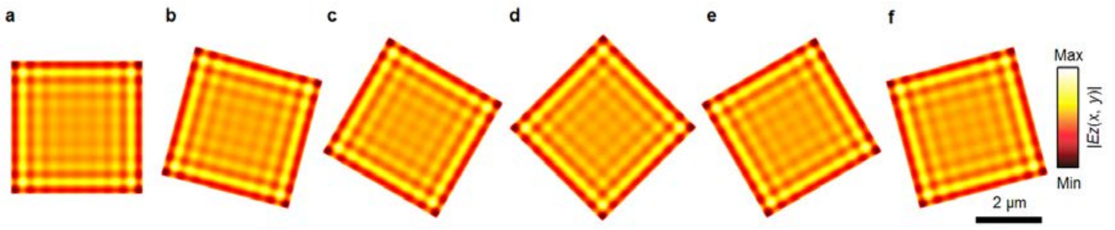

Supplementary Figure 6. The isotropic polariton distribution of square hBN nanocavities. The geometric dimensions in **a-f** are the same as the Fig. **2a** in main text.

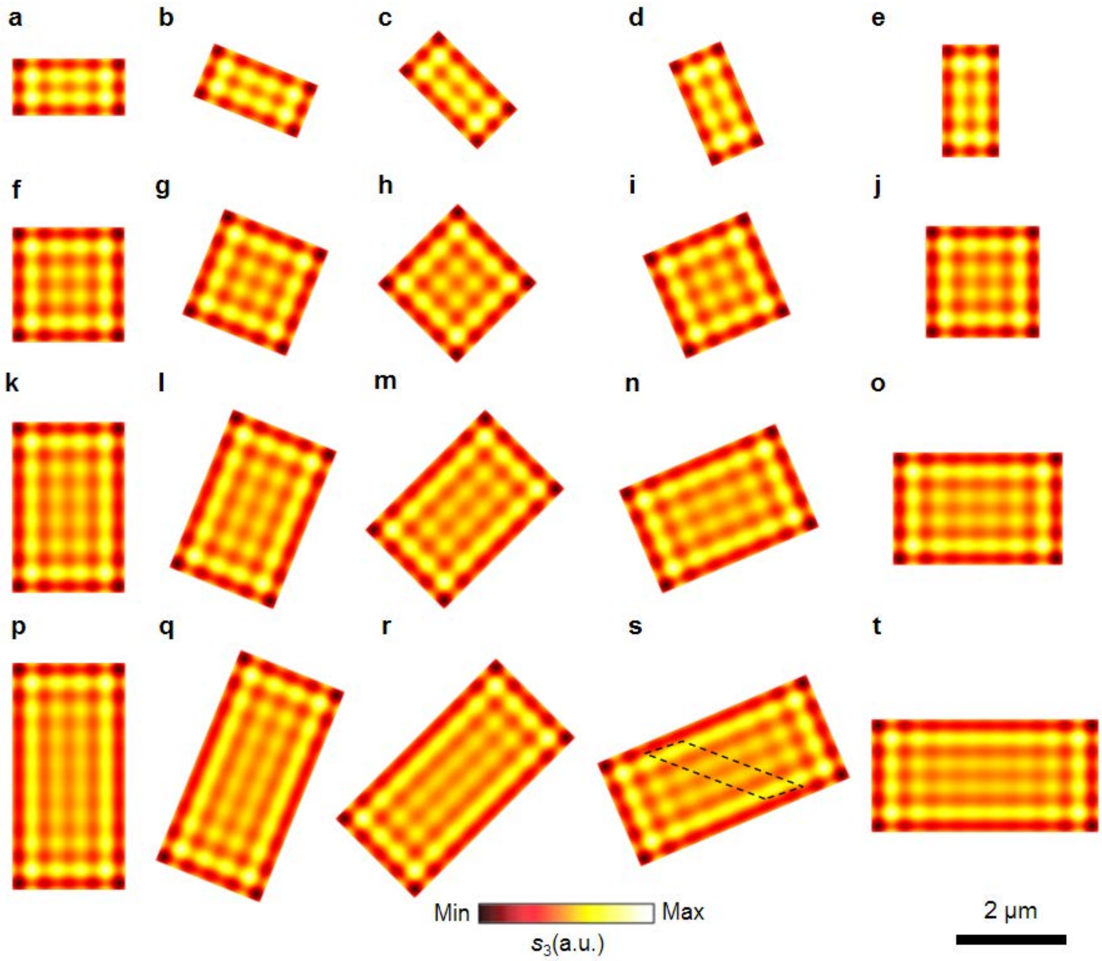

Supplementary Figure 7. The isotropic polariton distribution of hBN nanocavity with different edge aspect ratios. The geometric dimensions are the same as the **Fig. 3** in main text. The black dotted frame corresponds to the forbidden zone in **Fig. 3s**.

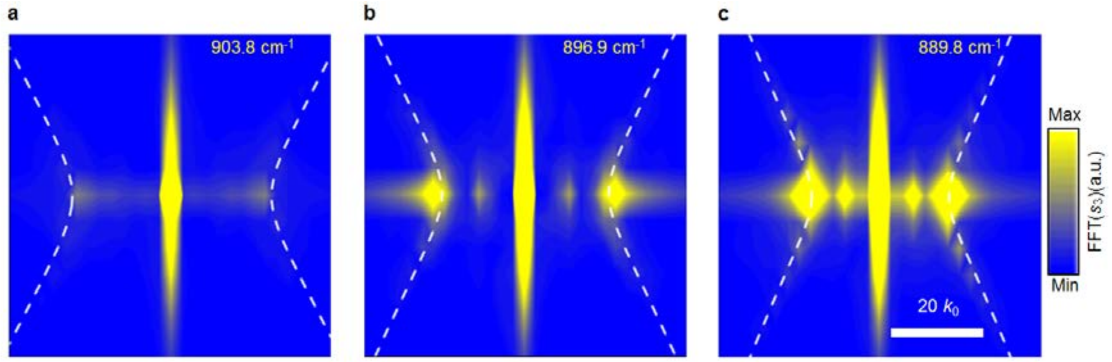

Supplementary Figure 8. **Iso-frequency contours of hyperbolic PhPs in large rectangle α-MoO_3 with different work frequencies. a-c**, absolute value of the Fourier transform $s(\omega)$ of the near-field images with different work frequency in the Fig 4h, respectively. Dot lines are the theoretical iso-frequency contours. k_0 is the free-space wavevector of light.

Supplementary Note 1. Reflection of hyperbolic PhPs at the edge of α-MoO_3 nanocavities

As shown in Supplementary Figure 2a and b and for an example, θ = 30°, the green and black dotted arrows illustrate the non-collinearity of wavevector $\mathbf{k}_i$ and Poynting vector $\mathbf{S}_i$ for a plane hyperbolic wave to the edge 1 in square α-MoO_3 nanocavity. The incident and reflected momenta $\mathbf{k}_i$ and $\mathbf{k}_e$ (green solid arrow) are antiparallel $\mathbf{k}_e = -\mathbf{k}_i$ and perpendicular to the edge (considered as a mirror).[1] However, due to the hyperbolic isofrequency contour (Supplementary Figure 2a), the incident Poynting vector $\mathbf{S}_i$ (perpendicular to hyperbolic isofrequency contour) is not parallel to wavevector $\mathbf{k}_i$. For the same reason, the reflected Poynting vector $\mathbf{S}_e$ (black solid arrow) is not parallel to $\mathbf{k}_e$ but antiparallel to $\mathbf{S}_i$ (see Supplementary Figure 2b). Consequently, both the wavefront and energy are reflected back to the source by the edge 1, although the energy flow is not perpendicular to the edge 1 (see Supplementary Figure 2b). The edge can originate from a groove (Ed1-Ed4 in Fig. 1c) or from an edge of the pattern (Figs. 2-4). But for the edge 2, θ = 60°, due to the dispersion bound, as shown in Supplementary Figure 2a, the incident $\mathbf{k}_{ip}$ should be huge to intersect with the isofrequency contour, and the corresponding polariton wavelength $\lambda_p = 2\pi/k_{ip}$ is too small to be observed. Therefore, the fringes cannot be observed when θ is 60° and more (60° ~ 90°, Ed5-Ed7 in Fig. 1c and Supplementary Figure 2b,). Here, we note, in s-SNOM, a metallic AFM tip is used to launch the polaritons when illuminated by a focused infrared light source; this tip then scatters and collects the polariton signal. In our measurement, the metallic tip, sweeping from one position to the other, can in principle launch all hyperbolic branches. However, the presence of the edge with an orientation angle limits that most power as reflected and returned to the tip, which we measured, is the polariton parallel to the edge.

Supplementary Note 2. Isotropic PhPs in triangular hBN nanocavities and edge-tailored PhPs in triangular α-MoO_3 nanocavities

As shown in Supplementary Figure 3a, in comparison with Fig. 1e, fringes are different in graphene[2] and hBN triangular nanocavities (Supplementary Figure 3a, named Tr1h-Tr5h), as the coaxial triangle fringes ($\mathbf{k}_e = \mathbf{k'}_e$) of polaritons due to the indiscriminate propagation and reflection at all edges. Both the graphene and hBN are an in-plane isotropic polaritonic material with the same hexagonal crystal structure; indeed, the in-plane permittivity components have the same value and sign.[3, 4] Furthermore, when the direction of the triangle bottom edge parallel to the [001] crystal direction in Supplementary Figure 3b (named as Tr’1-Tr’5), as the angles between adjacent sides of the series of triangles with respect to the [001] direction is less than 60°(Tr’1-Tr’3), the hyperbolic PhPs fringes are parallel to the bottom edge of the α-MoO_3 triangle nanocavities. However, as the angle approaches 30° (Tr’5), the hyperbolic PhP fringes parallel to the edges of the adjacent sides appear while the reflected wavevector $\mathbf{k}_e$ from bottom edge smaller than $\mathbf{k'}_e$ ($\mathbf{k}_e < \mathbf{k'}_e$) from the edges of the adjacent sides, which further reveals the effect of edge tailoring and directional guiding.

Supplementary Note 3. Calculated near-field amplitude images of triangular and rectangle α-MoO_3 nanocavities

The full-wave simulation with finite-difference time-domain method is performed by the commercial software Lumerical FDTD (2017b, http://www.lumerical.com/tcad-products/fdtd/). To obtain the polariton fringe, we use the plane-wave excitation, with the linear polarization angles dependent on the edge orientation to ensure all modes are launched, to excite the PhP nanocavity and the monitor at 20 nm top of the α-MoO_3 sample is used to record the PhPs. For rectangular shape, we use two linearly polarized light with the polarization directions along the edges, with the same

amplitude and under the normal incidence. For triangle shape, we use two linearly polarized light along *x* and *y* directions. In this simulation, although no AFM tips launching and collecting the polaritons have been included into our simulations and we do note the relationships between experimentally measured fields and excited polaritons via the plane waves are nontrivial, the numerically simulated polariton fringe patterns (Supplementary Figure 4 and Supplementary Figure 5) could help to understand the formation of PhPs within α-MoO_3 nanocavities in experiments. Indeed, the field distribution in Supplementary Figure 4 agrees with that in Fig. 1**e** and Supplementary Figure 3b, and the field distribution in Supplementary Figure 5 agrees with that in Fig. 3**k-o** in main text. The observations support the main discussion on the edge effect in main text, which shows the correlated number of fringes.

Supplementary Note 4. Numerical distributions of in-plane isotropic PhPs in hBN nanocavities with similar shapes

To compare our results of hyperbolic PhPs in the out-of-hyperbolic hyperbolic and in-plane isotropic polar van der Waals materials, we performed the numerical calculations in hBN nanocavity with the same geometric parameters. To model such nanocavity, the final polariton distribution results from the interference of the incident light that are focused on metallic AFM tips and the reflected polariton signals from all the edges, following the similar cases in plasmonics.[2, 5] Thus, the final field distribution can be written as $E(\mathbf{r}) = E_{\mathrm{inc}}(\mathbf{r}) + \sum_{\mathrm{i}} E_{\mathrm{r,i}}(\mathbf{r})$. Here, $E_{\mathrm{inc}}(\mathbf{r})$ denotes the incident field at the position of $\mathbf{r}$, which in our model is assumed to be normalized as 1. Besides, $E_{\mathrm{r,i}}(\mathbf{r}) = \mathrm{R_i e^{-i(k_{tr}-ik_{ti})\rho_i}}$, where $R_{\mathrm{i}} = R_0 \mathrm{e^{i\Phi_0}}$ denotes the reflection coefficient from the i-th edges, $k_{\mathrm{tr}} - \mathrm{i}k_{\mathrm{ti}}$ is the in-plane wavevector of hyperbolic PhPs in hBN, and ρ_{i} denotes the distance between the position $\mathbf{r}$ and i-th edge. In our calculations, we simply assume that the thickness of hBN is 200 nm, the working frequency is 1420 cm^{-1}, and the substrate is SiO_2. Thus, $k_{\mathrm{tr}} = \frac{2\pi}{950}\ \mathrm{rad\,nm^{-1}}$. The

damping ratio k_{ti}/k_{tr} is assumed 0.15. We also assume that R_0 is 1 as the result of small scattering due to the large momentum mismatch of free-space photon and polaritons, and the phase shift at the reflection interface is 1.5π. We do note those approximate values depend on the materials properties such as the richness of isotope. But our calculations effectively provide the polariton distributions of in-plane isotropic polariton fringes as shown in Supplementary Figure 3a and Supplementary Figures 6-7 below, which are largely different from our reported polaritonic distributions in nanocavities made of in-plane hyperbolic α-MoO_3. One dramatic conclusion that can be easily drawn after the comparison of the α-MoO_3 nanocavity is that the polariton fringes parallel to all edges exist regardless of the orientations in hBN nanocavity, which indicates the important role play of in-plane hyperbolicity of α-MoO_3.

Supplementary Note 5. Lifetimes of hyperbolic PhPs in large rectangle α-MoO_3 with different edge aspect ratios

The lifetimes of PhPs in different α-MoO_3 nanocavities were calculated along the [100] crystal direction using $\tau = L/v_g$,[6, 7] where the propagation length L was obtained by fitting a line scan of the near-field amplitude image and the group velocity v_g was extracted by fitting the dispersion relation curve and taking its first derivative ($v_g = \partial\omega/\partial k$). We first multiplied the experimental line profiles by a factor of $x^{1/2}$ to compensate the circular-wave geometrical spreading of the PhPs. Assuming a damped sine decay, we then fitted the obtained profile according to the following Supplementary Equation 1.

$$y = y_0 + A\mathrm{e}^{-\frac{x}{t_0}} \sin\left(\pi \frac{x-x_c}{w}\right), A > 0, w > 0, t_0 > 0 \quad (1)$$

In the fitting process, a Levenberg-Marquardt iteration algorithm was applied until a full convergence $(\Delta\chi^2 \leq 10^{-9})$. Finally, we extracted the propagation length as L = t_0.

The figure of merit (FOM) of PhPs in α-MoO_3 nanocavities with different edge aspect ratio were calculated along the [100] crystal direction using tunable frequency. The FOM defined in Supplementary Equation 2.

$$Q = \mathrm{Re}(\omega)/\mathrm{Im}(\omega) \tag{2}$$

can be used to characterize the loss features of polariton, where $\mathrm{Re}(\omega)$ is the center frequency and $\mathrm{Im}(\omega)$ is proportional to the fitted half-width at half-maximum of the spectral line widths extracted from the FFT of the s-SNOM linescans.[8]

Supplementary References

1. Ma, W. et al. In-plane anisotropic and ultra-low-loss polaritons in a natural van der Waals crystal. Nature **562**, 557 (2018).
2. Zheng, Z. B. et al. Tailoring of electromagnetic field localizations by two-dimensional graphene nanostructures. Light: Sci. Appl. **6**, e17057 (2017).
3. Caldwell, J.D. et al. Sub-diffractional volume-confined polaritons in the natural hyperbolic material hexagonal boron nitride. Nat. Commun. **5**, 5221 (2014).
4. Dai, S. et al. Tunable phonon polaritons in atomically thin van der Waals crystals of boron nitride. Science **343**, 1125-1129 (2014).
5. Gerber, J.A., Berweger, S., O'Callahan, B.T. & Raschke, M.B. Phase-Resolved Surface Plasmon Interferometry of Graphene. Phys. Rev. Lett. **113**, 055502 (2014).
6. Wu, Y. et al. Chemical switching of low-loss phonon polaritons in α-MoO_3 by hydrogen intercalation. Nat. Commun. **11**, 2646 (2020).
7. Woessner, A. et al. Highly confined low-loss plasmons in graphene-boron nitride heterostructures. Nat. Mater. **14**, 421-425 (2015).
8. Giles, A.J. et al. Ultralow-loss polaritons in isotopically pure boron nitride. Nat. Mater. **17**, 134 (2018).
